# Comparison of sensory quality perceptions of gluten-free cookies evaluated by celiac and non-celiac people

**DOI:** 10.3389/fnut.2025.1683571

**Published:** 2025-11-19

**Authors:** Daria Musiienko, Lenka Kouřimská, Diana Chrpová

**Affiliations:** Department of Microbiology, Nutrition and Dietetics, Faculty of Agrobiology, Food and Natural Resources, Czech University of Life Sciences Prague, Prague, Czechia

**Keywords:** celiac disease, non-celiac gluten sensitivity, gluten-free products, consumer acceptance, food sensory analysis, bakery products

## Abstract

Celiac disease affects about 1% of the population, requiring a strict gluten-free diet. Despite the growing market for gluten-free products, their sensory quality remains a challenge. Since celiacs must follow a gluten-free diet and cannot consume all cereal products like non-celiacs, such a limiting sensory experience could affect the sensory quality perception of the same cereal product. Therefore, this study compared how individuals with and without gluten-related disorders perceive the sensory properties of gluten-free biscuits. Sensory profile of three biscuit types (made from coconut shavings, buckwheat flour, and a gluten-free mix) were evaluated by 100 participants, including 48 individuals with celiac disease or non-celiac gluten intolerance, and 52 people without no dietary restrictions related to gluten. All data were organized and pre-processed in Microsoft^®^ Excel. The distribution of responses was examined using the Shapiro–Wilk test, which indicated deviations from normality. Consequently, non-parametric methods were applied: the Kruskal–Wallis test with Nemenyi *post-hoc* comparisons to analyze differences among biscuit types, and the Mann–Whitney U test to compare evaluations between participants with and without gluten-related disorders. Pearson's correlation coefficient was used to assess relationships among sensory attributes. Results showed no significant differences between people with glute-free diet and people without this restriction in majority of evaluated descriptors. Concerning the samples, the coconut-based biscuit received the highest ratings, while the buckwheat-based biscuit scored lowest. The results suggest that sensory evaluations of gluten-free products can be conducted by individuals without gluten-related disorders, as long-term adherence to a gluten-free diet does not significantly affect sensory perception.

## 1 Introduction

Celiac disease, which affects approximately 1% of the world's population, is an autoimmune disease in which gluten consumption leads to a reversible inflammatory process in the lining of the small intestine in affected individuals. This process causes acute symptoms such as diarrhea, constipation, bloating, nausea and vomiting (1). The only effective treatment for celiac disease is a lifelong gluten-free diet (2). Continued gluten intake in patients with celiac disease exacerbates clinical symptoms, further damages the gut, and increases the risk of developing cancers such as small bowel adenocarcinoma, esophageal cancer, melanoma, and non-Hodgkin's lymphoma (3). To achieve optimal outcomes, it is essential to eliminate gluten from the diet.

The number of consumers of gluten-free products is steadily increasing for a variety of reasons, including improved diagnosis of celiac disease (CD) and non-celiac gluten intolerance (NCNL). NCNL is a condition in which the consumption of gluten causes both intestinal and extra-intestinal symptoms such as abdominal pain, bloating, diarrhea, constipation and chronic fatigue. A gluten-free diet is also recommended for people with NCNL (4).

The growing interest in gluten-free foods has led to significant growth in this market segment, which was valued at USD 19.1 billion in 2022 and is expected to grow to USD 30.5 billion by 2028 (5). The growth of the gluten-free products (BLP) market is also fueled by the belief that gluten-free diets are healthier and more effective for weight management (6, 7). It is estimated that 25 % of the U.S. population consumes BLP, which far exceeds the number of Americans with celiac disease (8). However, studies report that removing gluten from the diet does not have a beneficial effect on the health of people without celiac disease. On the contrary, it may put them at risk for macro- and micronutrient deficiencies (9). In addition, regular consumption of gluten-free products often increases the intake of sugar, salt, and fat, which are used to improve the taste and texture of these foods. This can negatively affect cardiometabolic risk factors such as obesity, serum lipid levels, insulin resistance, metabolic syndrome, and atherosclerosis (10).

Despite the rapid growth of the gluten-free market, the quality of these products remains a major challenge. One of the most critical factors influencing both the nutritional profile and sensory acceptance of gluten-free bakery items is the choice of flour. Since wheat gluten cannot be used, manufacturers rely on a wide range of alternative flours and starches, each with distinct technological properties. Understanding these differences is essential to explain the variability in consumer perception and to guide the development of products that are both nutritious and sensorially appealing.

Various types of gluten-free flours have been studied as alternatives to wheat, each with distinct nutritional and sensory properties. Rice flour is frequently used due to its mild flavor, hypoallergenic profile, and light texture, although it often requires additional binding agents to improve structure (11). Corn and tapioca starch are common in gluten-free mixes, contributing to lightness and volume, but they lack protein and dietary fiber (12). Buckwheat flour, while nutritionally valuable and high in protein, has a strong flavor and darker appearance that can negatively influence sensory acceptance (13). Other studies have highlighted the potential of amaranth, sorghum, and quinoa flours to enrich protein and micronutrient content, although their incorporation requires technological adjustments to maintain palatability (7, 14). The choice of flour is therefore critical in gluten-free bakery product development, influencing not only nutritional value but also consumer acceptance.

In addition to their low nutritional value, BLPs are also more expensive, costing approximately two to three times more than similar standard products (15). Nevertheless, sensory properties remain one of the most important factors in purchasing decisions. Research by do Nascimento et al. (16) showed that bread was the most desired product among people following a gluten-free diet, but participants were often dissatisfied with the quality of gluten-free products. The desire for bread with better sensory properties highlights the call for the development of better-quality bakery products. Other studies have also confirmed that BLPs generally taste worse than their gluten-free counterparts (17).

Although many gluten-free products still have quality issues, research is helping to improve both their sensory properties and nutritional value. For example, Darniadi et al. (18) studied biscuits made with zinc-enriched rice and found no significant differences in sensory characteristics compared to regular biscuits. However, these biscuits had higher zinc and iron content. Cervini et al. (19) used resistant starch to slow carbohydrate absorption without negatively affecting sensory quality. More recent studies have tested ingredients like inulin and flaxseed powder (20). Collectively, these findings suggest that it is possible to develop gluten-free bakery products that meet both nutritional and sensory expectations.

Despite these advances, consumer research continues to report lower acceptance of gluten-free products compared to their wheat-based counterparts (16, 17). Efforts to improve flavor and texture through hydrocolloids, proteins, or fiber-rich ingredients have shown potential (19, 20). However, most sensory evaluations in these studies were conducted with assessors who had no dietary restrictions, leaving open the question of whether long-term adherence to a gluten-free diet alters sensory perception. To date, only a limited number of studies have directly compared the responses of celiac and non-celiac consumers (21), highlighting a gap in the literature.

This gap is particularly relevant because the target consumers of gluten-free products are individuals who must follow a strict gluten-free diet for medical reasons. Their sensory expectations may differ from those of healthy individuals, as long-term exclusion of gluten-containing foods prevents direct sensory comparison and may shape preference patterns over time. Therefore, understanding how celiac and non-celiac consumers evaluate gluten-free bakery products is essential for guiding product development that aligns with both sensory expectations and health needs.

Sensory evaluation of gluten-free products is often carried out by assessors who have no dietary restrictions. However, it is also important to conduct research that focuses specifically on the target group—people who consume gluten-free products not by choice but for health reasons. These people must follow a gluten-free diet and may have a limiting sensory experience which could affect their perception of the sensory quality of cereal products.

Therefore, the aim of this study was to investigate the sensory properties and perception of three types of gluten-free biscuits prepared from coconut shavings, buckwheat flour, and a ready-made gluten-free mix. By including both individuals with celiac disease or gluten intolerance and healthy consumers, this research addresses a gap in previous sensory studies and provides insights into whether dietary experience influences the perception of gluten-free bakery products.

## 2 Materials and methods

### 2.1 Sample preparation

**Figure 1:**
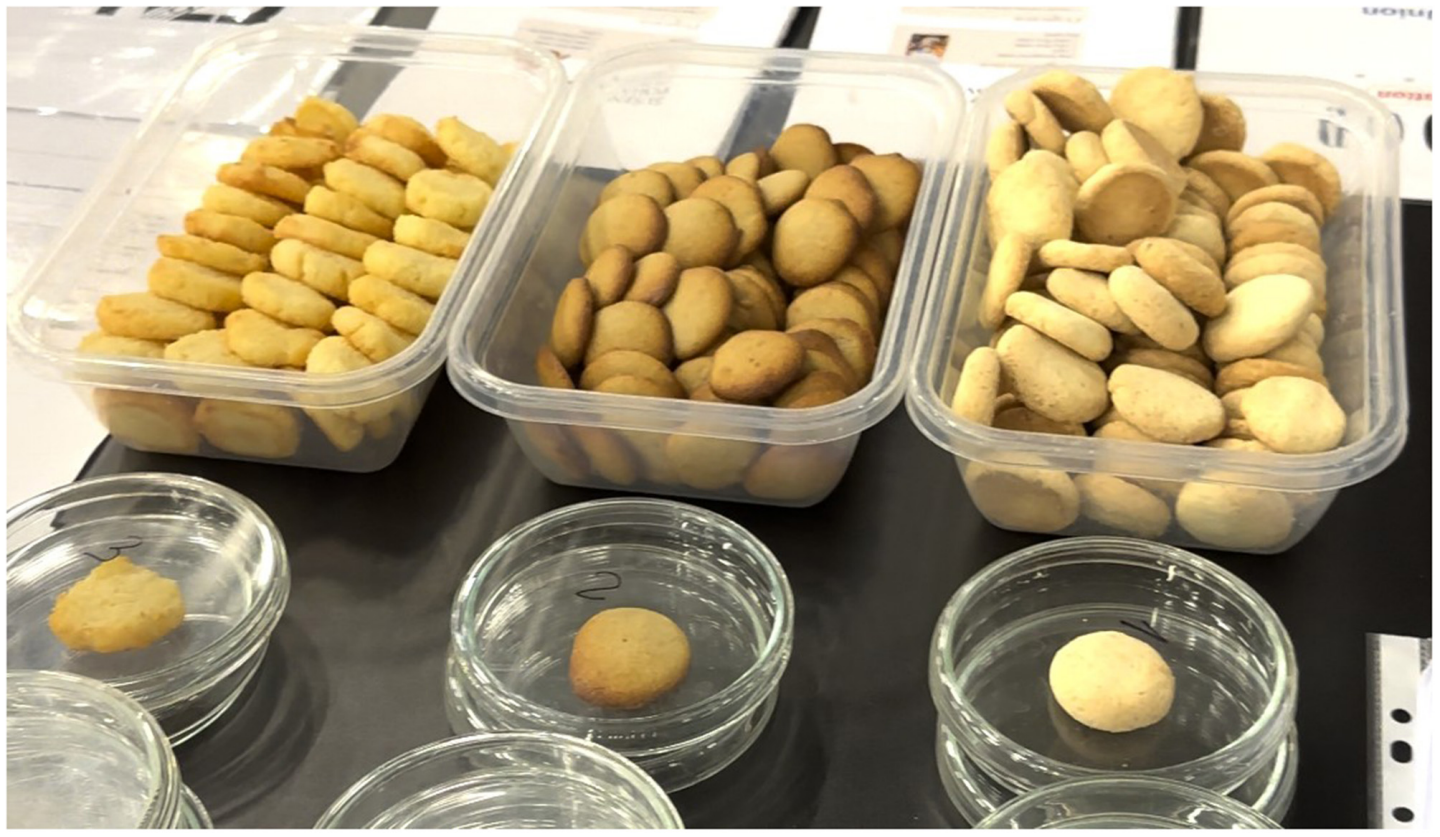
Visual appearance of the three types of gluten-free cookies evaluated in this study.

**Figure 2:**
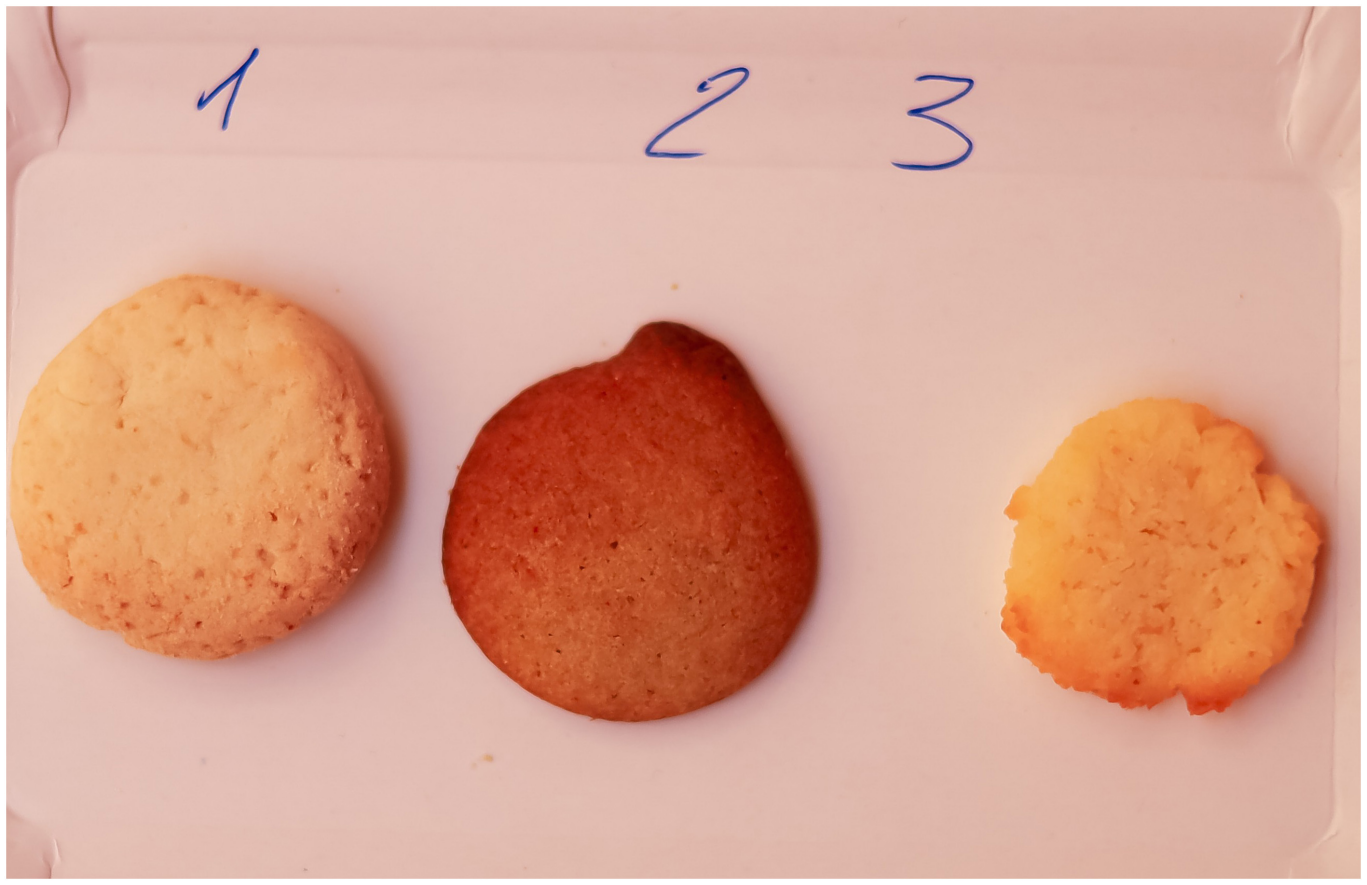
Detailed visual presentation of the three types of gluten-free biscuits.

The samples were prepared in the specialized kitchen of the gastronomic facility at the Czech University of Life Sciences in Prague, in the Agricultural Processing Training Centre. All ingredients were mixed to form a dough. The dough was then spooned into small lumps (about 10 g), which were placed on a baking sheet and baked. An electric combination oven (iCombiPro, Rational AG, Germany) was used in the “muffins/tea pastry” mode at 167 °C for 8 min. The main ingredients were butter (Jihočeské máslo 82%, Madeta, Czech Republic), sugar, eggs, raising agent (Baking powder, Dr. Oetker, Germany) and honey (Ceský květový med luční, Medokomerc, Czech Republic). Alternative flours have been used as a substitute for wheat flour in the biscuit recipe: buckwheat flour (Gluten-free buckwheat flour 400 g, Green Apotheke, Czech Republic), gluten-free cake mix (Směs na prípravu koláču bez lepku 1 kg, Nutrifree, Czech Republic) and grated coconut (Kokos Fein Geraspelt 200 g, K-Classic, Germany). The complete composition of the cookies recipes is given in Tables 1–3. The visual appearance of the three types of gluten-free cookies evaluated in this study is presented in Figure 1, which was used as a starting point for the sensory analysis. Additionally, a close-up photograph illustrating the surface texture (Figure 2) to provide a clearer visual comparison of their appearance.

**Table 1:** Cookies made from gluten-free flour mix.

**Ingredients**	**Amount**
^*^Gluten-free mix	150 g
Butter	65 g
Honey	25 g
Sugar	25 g
Egg	1 pc

^*^Gluten-free Cake Mix 1 kg, Nutrifree, Composition: Corn starch, rice flour, tapioca starch, sugar, plant fiber, thickener: guar gum and E464, salt, natural flavor., Nutritional values per 100 g: Energy value 1,463 kJ/345 kcal, fat 0.1 g (of which saturated 0.1 g), carbohydrates 83.9 g (of which sugars 1.2 g), fiber 2.8 g, protein 0.6 g, salt 0.4 g.

**Table 2:** Cookies made from Buckwheat flour.

**Ingredients**	**Amount**
^*^Buckwheat flour	70 g
Butter	50 g
Honey	25 g
Sugar	25 g
Egg	1 pc
Leavening agent	5 g

^*^Gluten-free Buckwheat Flour 400g, Green Apotheke, Composition: Ground hulled buckwheat. Nutritional values per 100 g: Energy value 1,428 kJ/343 kcal, fat 1.9 g (of which saturated fatty acids 0.4 g), carbohydrates 81.4 g (of which sugars 0.0 g), fiber 3.7 g, protein 10.5 g, salt < 0.1 g.

**Table 3:** Cookies made from grated coconut.

**Ingredients**	**Amount**
^*^Grated coconut	90 g
Butter	40 g
Sugar	25 g
Egg	1 pc

^*^Coconut Fein Geraspelt 200g, K-Classic, Composition: Grated coconut. Nutritional values per 100 g: Energy value 2,777 kJ/664 kcal, fat 65 g (of which saturated fatty acids 58 g), carbohydrates 6.4 g (of which sugars 6.4 g), fiber 21 g, protein 5.6 g, salt 0 g.

### 2.2 Sensory analysis

The study protocol was reviewed and approved by the Ethics Committee in Prague (File reference number: 01/2024, April 18, 2024). All participants were informed about the procedure and potential risks. A laboratory certificate confirming gluten content was available; only the buckwheat flour cookies contained trace amounts of gluten, which did not exceed the permissible levels for gluten-free products.

The sensory evaluation was conducted in two stages: at the 19th Celiac Forum – Gluten-Free Prague Expo 2024 and at the “LII. CzechFoodChem 2024” conference. Both sessions took place during daytime hours (approximately between 9:00 and 18:00) in exhibition and conference halls rather than individual sensory booths, under artificial lighting and without controlled room temperature. The conditions were consistent for all participants, and water was provided as a cleanser between samples.

A total of 100 individuals participated in the evaluation, including 4 with non-celiac gluten sensitivity, 44 with celiac disease, and 52 healthy individuals without dietary restrictions. The number of participants in the sensory evaluation (*n* = 100) is consistent with recent studies on gluten-free bakery products, which typically recruit 80–100 untrained consumers and are sufficient to detect differences in consumer perception across groups. For example, a recent study comparing gluten-free and gluten-containing chocolate cookies involved 84 consumers in sensory testing (22). Importantly, recruiting individuals with medically diagnosed celiac disease or gluten intolerance is particularly challenging, and nearly half of our participant group (*n* = 48) represented this population, a scale rarely reported in consumer sensory research.

The gender distribution of participants (86 females and 16 males) reflects the well-documented predominance of females among individuals with celiac disease, with female-to-male ratios commonly reported around 2–2.5:1 (23). The participants' ages ranged from 9 to 80 years, covering a broad consumer spectrum. Duration of adherence to a gluten-free diet among individuals with celiac disease or non-celiac gluten sensitivity varied from 6 months to 18 years.

Cookie samples were served in Petri dishes, labeled with numerical codes to ensure anonymity. Sensory testing was carried out using unstructured continuous 100 mm graphical scales (a horizontal line with a low rating associated with the left-hand and of the line and a high rating at the right-hand end of the line) for seven descriptors: overall appearance, color acceptability, aroma acceptability, texture acceptability, taste acceptability (0% = unpleasant, 100% = pleasant), sweetness intensity, and off-flavor/aftertaste intensity (0% = imperceptible, 100% = strong). After data collection, the distance (in millimeters) from the left anchor point (“unpleasant” or “imperceptible”) to the participant's mark was measured with a ruler and recorded as a numerical score ranging from 0 to 100. These values were then entered into a digital spreadsheet for statistical processing. To guide the evaluation, participants were explicitly asked: “How acceptable is the overall appearance, color, aroma, texture, and taste of the sample? How intense is its sweetness? How intense is its off-flavor/aftertaste?”

The inclusion of sweetness intensity and off-flavor intensity was based on their established role as critical determinants of consumer acceptance of bakery products. Prior studies have demonstrated that excessive sweetness can negatively affect hedonic responses, whereas the perception of undesirable aftertastes is a frequent cause of product rejection (24, 25). An example of the evaluation form is provided in Appendix 1.

### 2.3 Statistical analysis

All data were organized and pre-processed in Microsoft^®^ Excel (version 16.89.1). The distribution of responses was examined using the Shapiro–Wilk test, which indicated deviations from normality. Therefore, non-parametric methods were applied for subsequent analyses.

Comparisons among the three biscuit types were conducted using the Kruskal–Wallis test, with Nemenyi *post-hoc* pairwise comparisons to identify specific group differences. To evaluate potential differences between participants adhering to a gluten-free diet and those without dietary restrictions, the Mann–Whitney U test with a two-tailed hypothesis was employed. A significance level of α = 0.05 (95 % confidence level) was applied throughout all analyses.

Relationships among sensory attributes were assessed using Pearson's correlation coefficient, as all variables were measured on continuous scales.

Statistical tests were performed using Microsoft^®^ Excel together with validated online statistical tools: Shapiro–Wilk normality test (StatsKingdom, www.statskingdom.com), Kruskal–Wallis test (Astatsa, www.astatsa.com), and Mann–Whitney U test (Social Science Statistics, www.socscistatistics.com).

## 3 Results

### 3.1 Sensory evaluation without considering gluten-related factors

The sensory evaluation was conducted to test the hypothesis that the three biscuit formulations differ significantly in their sensory characteristics (H0: no differences among formulations; H1: at least one formulation differs). According to the Kruskal–Walli's test, significant differences were observed among the three biscuit samples across all evaluated sensory attributes, reflecting deviations from normality in the data distributions. *Post-hoc* Nemenyi comparisons indicated that the buckwheat-based Sample (Sample 2) consistently received lower scores for overall appearance, color acceptability, aroma acceptability, taste, and sweetness intensity compared to the gluten-free flour mix biscuits (Sample 1) and the coconut-based biscuits (Sample 3). The coconut-based Sample (Sample 3) generally achieved the highest ratings, particularly for pleasantness of color, aroma, texture, and taste. No significant differences were observed between samples 1 and 3 for overall appearance and sweetness intensity, while minor differences were detected for other attributes, such as color and taste. For intensity of off-flavor, Sample 3 was rated slightly higher than Sample 2, with no differences relative to Sample 1. These results highlight the influence of ingredient composition on sensory perception, with the coconut-based formulation emerging as the most preferred. More detailed statistical results, including Kruskal–Wallis χ^2^, degrees of freedom, *p-*values, and *post-hoc* pairwise comparisons, are presented in Table 4.

**Table 4:** Kruskal–Wallis test results and Nemenyi *post-hoc* comparisons for sensory attributes of gluten-free cookies.

**Parameter**	**χ^2^**	**df**	***p*-value**	**Significant differences (Nemenyi *post-hoc*)**
Overall appearance	29.53	2	< 0.001	2 < 1 and 3, 1 = 3
Pleasantness of color	53.94	2	< 0.001	2 < 1 and 3, 1 < 3
Pleasantness of smell	47.46	2	< 0.001	2 < 1 and 3, 1 < 3
Pleasantness of texture	25.61	2	< 0.001	3 > 1 and 2, 1 = 2
Pleasantness of taste	37.03	2	< 0.001	2 < 1 and 3, 1 < 3
Intensity of sweet taste	28.07	2	< 0.001	2 < 1 and 3, 1 = 3
Intensity of off-flavor	6.83	2	0.033	3 > 2, 1 = others

### 3.2 Correlation analysis

Pearson's correlation analysis was conducted to examine the linear relationships between sensory parameters, as all variables were measured on continuous scales. Correlation coefficients (r) were interpreted according to established guidelines, where r = 0.10, r = 0.30, and r = 0.50 were recommended to be considered small, medium, and large in magnitude, respectively (26).

The correlation analysis was conducted to test the hypothesis that sensory attributes of gluten-free cookies are interrelated rather than independent. Specifically, the null hypothesis (H0) assumed no significant linear relationships between evaluated sensory parameters, whereas the alternative hypothesis (H1) assumed the presence of significant positive or negative correlations.

The analysis revealed a large positive correlation between color acceptability and overall appearance (r = 0.73), indicating that biscuits rated higher in color were also perceived as visually more appealing. Large positive correlations were also observed between color acceptability and aroma acceptability (r = 0.59), texture acceptability (r = 0.50), and taste acceptability (r = 0.55), as well as between taste acceptability and both texture acceptability (r = 0.65) and aroma acceptability (r = 0.56). These relationships suggest that improvements in one sensory attribute tended to coincide with higher ratings in related attributes. Conversely, aftertaste intensity showed a medium negative correlation with taste acceptability (r = −0.30), suggesting that stronger off-flavor may reduce overall taste satisfaction.

Overall, these findings emphasize the key role of ingredient composition in shaping sensory perception of gluten-free biscuits, with coconut-based biscuits consistently emerging as the most preferred, likely due to their favorable combination of flavor, texture, and aroma. Detailed correlation coefficients for all sensory parameters are provided in Table 5.

**Table 5:** Correlation between sensory parameters.

**Parameter**	**Overall appearance**	**Color acceptability**	**Aroma acceptability**	**Texture acceptability**	**Taste acceptability**	**Sweetness intensity**	**Off-flavor intensity**
Overall appearance	1.000000	0.727407	0.467529	0.461854	0.448677	0.320751	−0.038470
Color acceptability	0.727407	1.000000	0.592933	0.502676	0.553811	0.354933	−0.118544
Aroma acceptability	0.467529	0.592933	1.000000	0.420250	0.559810	0.338403	−0.226956
Texture acceptability	0.461854	0.502676	0.420250	1.000000	0.645635	0.190964	−0.218049
Taste acceptability	0.448677	0.553811	0.559810	0.645635	1.000000	0.384583	−0.304642
Sweetness intensity	0.320751	0.354933	0.338403	0.190964	0.384583	1.000000	0.016736
Off-flavor intensity	−0.038470	−0.118544	−0.226956	−0.218049	−0.304642	0.016736	1.000000

Red correlation coefficients indicate significant relationship between tested parameters.

### 3.3 The effect of evaluator groups

The analysis of evaluator groups was based on the hypothesis that adherence to a gluten-free diet may influence sensory perception. The analysis of evaluator groups was based on the hypothesis that adherence to a gluten-free diet may influence sensory perception. The null hypothesis (H0) assumed no differences in sensory ratings between participants with gluten-related disorders and those without such restrictions. The alternative hypothesis (H1) assumed that the two groups would differ significantly in their evaluation of at least one sensory attribute.

To examine potential differences between participants adhering to a gluten-free diet and those without dietary restrictions, data were analyzed using the non-parametric Mann–Whitney U test with a two-tailed hypothesis, as the distributions of responses were not normal. The analysis revealed a small number of statistically significant group differences.

For Sample 1 (gluten-free flour mix), a significant difference was observed only in the parameter Intensity of off-flavor (z = −2.687, *p* = 0.007), with the gluten-free group rating this attribute higher (mean = 40) compared to the non-restricted group (mean = 24).

For Sample 2 (buckwheat flour), significant differences were identified in Overall appearance (z = −2.155, *p* = 0.032) and Intensity of off-flavor (z = −2.242, *p* = 0.025). In both cases, participants on a gluten-free diet provided higher ratings (means = 56 and 41, respectively) compared to the second group (means = 45 and 28, respectively).

For Sample 3 (coconut-based), a significant difference was observed in Overall appearance (z = −2.082, *p* = 0.038), where the gluten-free group rated the cookies higher (mean = 73) than participants without dietary restrictions (mean = 65).

Overall, although a few attributes differed significantly between groups, the magnitude of these differences was relatively small and did not alter the general finding that celiac and non-celiac participants evaluated the gluten-free cookies in a broadly similar manner. More detailed calculations, including mean values and index-based comparisons, are provided in Table 6.

**Table 6:** Mean sensory evaluation scores and standard deviations for gluten-free cookies.

**Sample**	**Overall appearance**	**Color acceptability**	**Aroma acceptability**	**Texture acceptability**	**Taste acceptability**	**Sweetness intensity**	**Off-flavor intensity**
**1 Y**	66.38 ± 21.89	67.96 ± 22.52	66.69 ± 26.75	53.44 ± 27.38	62.00 ± 26.10	58.65 ± 24.27	40.27 ± 29.81^a^
**1 N**	64.04 ± 21.75	62.31 ± 24.86	70.23 ± 16.22	55.62 ± 27.8	63.12 ± 23.81	55.65 ± 16.48	24.00 ± 23.70^b^
**2 Y**	56.21 ± 27.45^a^	53.91 ± 22.20	54.36 ± 28.36	55.28 ± 28.33	52.70 ± 26.77	39.98 ± 22.12	41.28 ± 28.36^a^
**2 N**	44.69 ± 21.62^b^	45.77 ± 23.64	55.92 ± 23.01	48.54 ± 23.72	51.96 ± 23.76	41.04 ± 18.32	28.31 ± 22.00^b^
**3 Y**	73.17 ± 22.07^a^	78.77 ± 18.51	79.29 ± 21.73	72.52 ± 26.80	73.63 ± 25.74	51.69 ± 25.15	32.96 ± 30.24
**3 N**	64.80 ± 20.17^b^	75.02 ± 16.38	78.82 ± 14.39	67.47 ± 22.10	75.14 ± 14.19	53.37 ± 18.54	18.08 ± 16.24

Y = evaluations by individuals with celiac disease or NCGL, N = evaluations by individuals without gluten-related disorders. Different indices in the same column show significantly different samples (*p* < 0.05).

## 4 Discussion

This study aimed to compare the sensory perception of gluten-free biscuits between individuals with celiac disease or gluten intolerance and those without dietary restrictions. The discussion interprets the main findings in light of previous research and highlights which product attributes most strongly influenced consumer acceptance. In particular, attention is given to the sensory properties of the formulations, as well as to potential differences between evaluator groups.

### 4.1 Sensory evaluation of formulations

The sensory analysis revealed significant differences between the three biscuit formulations. Sample 3 (coconut-based) consistently received the highest ratings for most sensory attributes, particularly for overall appearance, color, aroma, texture and taste acceptability. These findings align with recent studies showing that coconut-derived ingredients (flour, oil, and coconut-based sweeteners) can enhance consumer liking by contributing natural sweetness, characteristic aroma volatiles, and higher fat content that improves mouthfeel and texture (27–30). Our coconut-based cookies likely benefited from (i) lipid-driven flavor release and creamy mouthfeel, and (ii) caramel-like notes from coconut-derived sugars. Comparable acceptance gains were reported when coconut ingredients were used in GF cookies and cakes, where coconut oil/flour increased overall liking, aroma and sweet taste scores (28, 30). A trained-panel lexicon study comparing market GF vs. GC cookies also found that well-formulated GF cookies can cluster near GC products on key aroma/flavor and texture axes, supporting our high hedonic scores for Sample 3 (29). When coconut-based sweeteners were used in GF corn cookies, panels associated them with caramel and buttery aroma as well as caramel taste, providing a plausible mechanism for the higher color/appearance and aroma liking observed in our Sample 3 (28). Additionally, coconut flour and shredded coconut are known to improve texture, making products softer and more palatable (31). The high fat content of coconut (around 64%) contributes to a smooth mouthfeel and rich flavor, while its natural sweetness further enhances overall taste perception (32). Coconut flour is also high in fiber (4.48%−8.31%), which not only improves satiety but also adds a slight crunch, making the biscuits more enjoyable (33). Furthermore, the volatile compounds in coconut, such as lactones, contribute to its distinct aroma, associated with tropical and buttery flavors, further influencing consumer preference (34). However, despite these advantages, the use of coconut-based ingredients presents some challenges. The high fiber content can increase moisture retention, potentially making biscuits denser if not balanced with other ingredients (32). Additionally, the natural fat content can lead to a shorter shelf life due to oxidation risks (31). Moreover, coconut flour lacks gluten, which can affect the structural integrity of baked goods. To counteract this, binders like eggs or hydrocolloids are often necessary to maintain consistency (14).

In contrast, Sample 2 (buckwheat-based) received significantly lower ratings, particularly in terms of appearance, color acceptability, aroma, and taste. Buckwheat flour has a strong, distinctive flavor that can easily dominate the taste profile of baked goods. Its sensory characteristics vary depending on production methods, with processing techniques influencing both flavor and secondary product attributes (13). Literature consistently notes that buckwheat brings strong earthy and bitter notes, mainly linked to phenolic compounds and flavonoids, which can reduce consumer liking unless these flavors are mitigated through processing or flavor-masking strategies. Recent reviews and process-modification studies, for example, steam explosion treatment of Tartary buckwheat flour, report significant improvements in cookie sensory quality after reducing bitterness and harsh notes, which is consistent with our panel feedback (35, 36). This aligns with the findings of Yamsaengsung et al. (37), where buckwheat cookies were rated poorly, especially due to their greenish hue, which negatively affected appearance scores. In our study, the buckwheat cookies were darker brown, but they still received the lowest color acceptability scores. Moreover, textural issues were also observed. Yamsaengsung et al. (37) described buckwheat cookies as crumbly with a strong aftertaste, and similar characteristics were evident in our sample. However, Yamsaengsung et al. (37) found that adding 60% chickpea flour significantly improved the sensory scores of buckwheat cookies, particularly their texture. This suggests that combining buckwheat flour with alternative gluten-free flours or incorporating additional binding agents could mitigate its dominant sensory characteristics and enhance consumer acceptance. In addition, buckwheat biscuits were perceived as less sweet compared to other formulations, which may have further reduced taste acceptability, as insufficient sweetness is often associated with lower hedonic scores in cookies.

Sample 1 (a gluten-free mix of corn starch and rice flour) exhibited intermediate sensory characteristics, reflecting both advantages and limitations. Rice flour is a popular choice for gluten-free formulations due to its mild taste, neutral color, hypoallergenic properties, and digestible carbohydrates (11). Rice-based GF cookies are widely used as a baseline and are typically acceptable yet perceived as less flavorful and less rich unless complemented with fruit-based sweeteners or fibers. Recent studies with rice cookies sweetened with date paste or blended with green banana flour reported significant improvements in consumer liking and texture vs. plain rice controls, which mirrors our moderate scores for the rice sample (38, 39). Nonetheless, gluten-free formulations often struggle to achieve optimal texture, as the absence of gluten can result in a crumbly or dry consistency. To improve texture, additional binding agents, such as xanthan gum, psyllium husk, or flaxseed, are often used (12). Hydrocolloids modulate spread, hardness and moisture retention in GF cookies. Multiple studies report that increasing xanthan gum raises hardness, browning index and water activity while reducing spread, indicating the need for careful optimization; conversely, psyllium can improve structure and mouthfeel, but excessive levels may lower acceptance. These patterns align with our instrumental texture observations and panel notes (40).

### 4.2 Effect of evaluator groups

The findings of this study provide valuable insights into how individuals with celiac disease and gluten intolerance perceive gluten-free biscuits compared to those without dietary restrictions. Overall, the two groups showed remarkably similar evaluations: no significant differences were found in five out of seven descriptors, including taste acceptability. Differences emerged only for off-flavor intensity and overall appearance in Sample 1 and Sample 2, and for overall appearance in Sample 3. Off-flavor deserves particular attention, since even mild undesirable aftertastes can strongly reduce consumer liking and purchase intent. Interestingly, participants with gluten-related disorders tended to give higher ratings for off-flavor, suggesting that long-term consumers of gluten-free products may be more sensitive or attentive to this attribute, possibly due to their habitual exposure to such formulations. Nevertheless, these differences were relatively minor and did not change the overall conclusion that adherence to a gluten-free diet does not fundamentally alter sensory perception. This finding is consistent with previous research by Laureati et al. (21), who reported that individuals with celiac disease expressed similar sensory preferences to healthy consumers when evaluating gluten-free bread. At the same time, the study revealed considerable variability in individual ratings, as reflected by the high standard deviations. This variability indicates that consumer preferences are not uniform, and non-expert panels may generate a wide spread of responses. Consequently, larger and more diverse consumer groups should be recruited to ensure robust and reliable sensory results. From a practical perspective, this suggests that while recruiting evaluators with dietary restrictions is not strictly necessary for gluten-free product development, gathering input from a broad audience can provide more accurate insights into general consumer acceptance. Despite the lack of significant differences between the groups, the study revealed considerable variability in the ratings (as indicated by the high standard deviation). This variability suggests that consumer preferences are not uniform, and non-expert consumers may show a broader range of responses. This highlights the importance of gathering a larger sample of responses to ensure the robustness and reliability of sensory results. While specialized evaluators with dietary restrictions may not be necessary for product development, obtaining diverse feedback from a wider audience could provide more accurate insights into general consumer preferences.

## 5 Conclusion

This study demonstrated that individuals with celiac disease, non-celiac gluten intolerance, and those without dietary restrictions rated gluten-free biscuits similarly across most sensory attributes. These findings indicate that long-term adherence to a gluten-free diet does not significantly alter sensory perception, which has important theoretical implications for future sensory research. Specifically, the results support the validity of using mixed consumer panels in sensory testing of gluten-free products and contribute new evidence to the ongoing discussion on whether dietary experience influences hedonic responses.

From a practical perspective, the study highlights the critical role of ingredient composition in shaping consumer preferences for gluten-free bakery products. Coconut-based biscuits achieved the highest overall liking due to their favorable balance of flavor, texture, and aroma, suggesting that optimized lipid systems, natural sweeteners, and fiber combinations can significantly improve sensory quality. Conversely, buckwheat-based biscuits received the lowest acceptance, underlining the need for formulation adjustments such as combining buckwheat with milder flours, applying flavor-masking strategies, or adopting process modifications to reduce bitterness. The results also provide actionable insights for food manufacturers and product developers. Using targeted formulation strategies, such as integrating coconut-derived ingredients, carefully balancing hydrocolloids, and experimenting with natural flavor enhancers, can help narrow the sensory gap between gluten-free and gluten-containing products, ultimately improving consumer satisfaction and purchase intent.

This work also contributes to the literature by addressing an important gap: direct comparisons of celiac and non-celiac consumers in gluten-free sensory testing remain rare. Future research should expand the scope to other product categories, apply advanced instrumental analyses of flavor volatiles and texture, and explore consumer emotional responses alongside hedonic ratings. Such approaches could facilitate the development of evidence-based guidelines for optimizing gluten-free formulations and improving market competitiveness.

Nevertheless, several limitations of the present study should be acknowledged. First, the sample size was relatively small, and the inclusion of non-expert participants may have contributed to the high variability in ratings. Second, the study examined only a limited number of gluten-free biscuit formulations, which restricts the generalizability of the findings to other gluten-free products. Finally, the evaluation relied exclusively on sensory assessments, without instrumental analyses of flavor or texture, which could have provided additional objective insights.

Despite these limitations, the study provides valuable evidence that both consumers with and without gluten-related disorders evaluate gluten-free biscuits in a broadly similar way. It also underscores the importance of improving sweetness intensity and minimizing off-flavor as key strategies for enhancing product acceptance.

## Data Availability

The datasets presented in this study can be found in online repositories. The names of the repository/repositories and accession number(s) can be found below: https://data.mendeley.com/datasets/5rxxwfbk5x/1.
