# Supplementary figures and images for: Comparison of sensory quality perceptions of gluten-free cookies evaluated by celiac and non-celiac people
Source: Front Nutr. 2025 Nov 19;12:1683571. doi: 10.3389/fnut.2025.1683571 (PMC12673662; doi:10.3389/fnut.2025.1683571)

Appendix

Appendix 1. Sensory evaluation questionnaire for gluten-free cookies


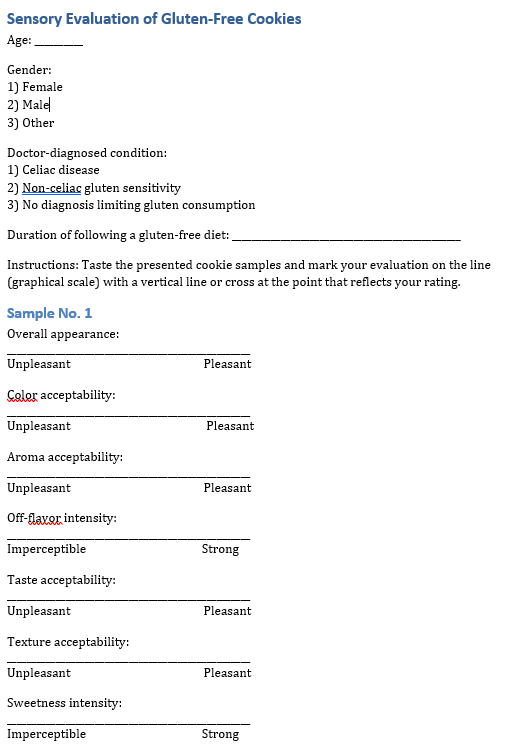

Supplement: Supplementary file 1 [file Data_Sheet_1.docx]
